# The precision of 12-month-old infants’ link between language and categorization predicts vocabulary size at 12 and 18 months

**DOI:** 10.3389/fpsyg.2015.01319

**Published:** 2015-08-31

**Authors:** Brock Ferguson, Mélanie Havy, Sandra R. Waxman

**Affiliations:** ^1^Department of Psychology, Northwestern University, Evanston, ILUSA; ^2^Department of Psycholinguistics, Université de Genève, GenevaSwitzerland

**Keywords:** word learning, categorization, infants, individual differences, vocabulary, longitudinal

## Abstract

Infants’ initially broad links between language and object categories are increasingly tuned, becoming more precise by the end of their first year. In a longitudinal study, we asked whether individual differences in the precision of infants’ links at 12 months of age are related to vocabulary development. We found that, at 12 months, infants who had already established a precise link between labels and categories understood more words than those whose link was still broad. Six months later, this advantage held: At 18 months, infants who had demonstrated a precise link at 12 months knew and produced more words than did infants who had demonstrated a broad link at 12 months. We conclude that individual differences in the precision of 12-month-old infants’ links between language and categories provide a reliable window into their vocabulary development. We consider several causal explanations of this relation.

## Introduction

Human infants are born with a preference for listening to language (Vouloumanos and Werker, 2007; Vouloumanos et al., 2010). They also link the sounds of language to core cognitive capacities (Vouloumanos and Waxman, 2014). One such link – between language and object categorization – is present in the first months of life and becomes increasingly precise over the first year. Here we ask: is there a relation between infants’ advances in the precision of this link and their advances in vocabulary development?

The link between language and object categorization is initially broad. In their first year, infants form object categories more successfully when listening to language than when listening to other sounds (e.g., tone sequences or backward speech; Balaban and Waxman, 1997; Fulkerson and Waxman, 2007; Ferry et al., 2010). During this time, object categorization is enhanced merely by the presence of language, and not necessarily from their focus on particular words: Even when infants cannot understand the words (for example, when the language signal has been filtered to mask which particular words are being said), listening to language still boosts their object categorization (Balaban and Waxman, 1997).

By 12 months, however, infants reach a turning point where what matters is not *whether* infants are listening to language but, more precisely, what is being said about each object. In particular, what matters is whether the *same* or *different* words are applied to a set of objects. Waxman and Braun (2005) demonstrated this newfound precision in the link between language and categorization using a novelty preference paradigm in which infants were familiarized to four distinct objects from a single category (either ANIMALS or TOOLS). What varied across conditions was whether infants heard the same word applied consistently to all members of the set (e.g., *Look at the keeto! Look at the keeto!…*) or a different word applied to each (e.g., *Look at the keeto! Look at the bookoo!…*). At test, infants were shown two novel objects simultaneously in silence – one that belonged to the same category as familiarization and one that belonged to a novel category. It was predicted that those infants who formed the category would show a preference for the novel object (Colombo and Bundy, 1983; Eimas and Quinn, 1994). They found that those infants who heard the same word applied consistently to all familiarization objects categorized successfully and preferred the novel object, but infants who heard a distinct word applied to each familiarization object performed at chance (Waxman and Braun, 2005). This documented that, by 12 months, infants track not only *which* objects and *which* words are presented, but also *how* the words and objects are paired (see also Smith and Yu, 2008). Specifically, when they hear the same label with each object, the labels highlight commonalities between them and thus facilitate their categorization (Waxman and Markow, 1995; Balaban and Waxman, 1997; Waxman and Booth, 2003; Fulkerson and Waxman, 2007; Ferry et al., 2010). Other studies suggest that when they hear a distinct label for each object, the labels highlight their differences and thus facilitate their individuation (Xu et al., 2005; Dewar and Xu, 2007; Feigenson and Halberda, 2008).

But what are the consequences of this increasingly precise link between language and categorization for early word learning? Does this more precise link coincide with an advantage in acquiring the meanings of words? We know that general categorization skill and vocabulary development are broadly correlated in infants’ second year (Gopnik and Meltzoff, 1987; Poulin-Dubois et al., 2008), and that, at least at 20 months, infants’ use of novel labels as guides to category formation also indexes vocabulary development (Nazzi and Gopnik, 2001). However, we do not yet know whether individual differences in the *precision* of infants’ link between language and categories can be traced to differences in the pace of early word learning.

To address this issue, we conducted a longitudinal study beginning with infants at 12 months of age – a turning point not only in the precision of the link to categories (Waxman and Braun, 2005) but also in vocabulary growth (Dale and Fenson, 1996). For each infant, we measured the precision of his/her link between language and object categorization at 12 months. We also measured the number of words in their lexicons at 12 months and again at 18 months. In addition to considering infants’ total vocabularies, we also teased apart two subsets: nouns and non-nouns. We hypothesized that infants’ performance on the object categorization task might be related more strongly to nouns (which refer to objects and object categories, especially for young infants) than non-nouns (which do not refer to object categories; Waxman, 2003). The longitudinal component of our design permitted us to ask whether the precision of an infants’ language-category link was related to their current and future advances in vocabulary development.

**FIGURE 1:**
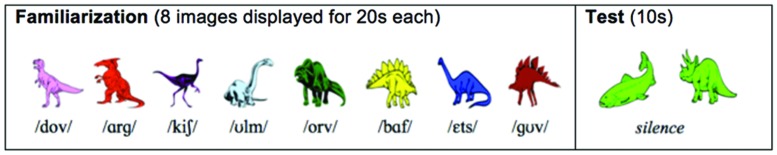
**A representative set of familiarization and test phase stimuli.** Each label was presented within the same sentence frame: “Look at the *[label]*! Do you see the *[label]*?”.

To measure infants’ precision in linking language to object categories, we adapted Waxman and Braun’s (2005) categorization task in which a different word was paired with each familiarization object (their Variable Word condition; see **Figure 1**). We used a set of stimuli for which the effect of consistently applying the same name had been previously established: For infants ranging from 3 to 12 months, infants who hear the same name consistently applied to these stimuli reliably form object categories (Fulkerson and Waxman, 2007). Here, however, we asked how infants fared when each familiarization object is named with a different word.

We predicted that these infants would fail, as a group, to form object categories (as in Waxman and Braun’s, 2005 original study). We also expected that there would be variability in this condition, with some infants categorizing in the context of the different labels (and thus showing a novelty preference) and other infants – those with a more precise link – not categorizing (and thus showing a chance or familiarity preference). Our goal was to use this variability to test the relation between the specificity of infants’ language-category link and their vocabulary growth. By examining vocabulary at both 12 and 18 months, we were able to assess the predictive power of infants’ language-category link during a period of especially rapid lexical growth (Dale and Fenson, 1996).

We reasoned as follows: If the precision of the language-category link at 12 months is related to infant vocabulary development, then infants who exhibit a precise link in our task should have larger vocabularies than those with a more broad link. Notice that this prediction runs counter to the perhaps more intuitive idea that infants who exhibit a broad link (that is, infants who form an object category even in the face of hearing different labels applied to each object) should have the larger vocabularies. This counter claim is certainly plausible. After all, both categorization and vocabulary learning draw on processing and memory skills shared by myriad other cognitive capacities (Gopnik and Meltzoff, 1987; Nazzi and Gopnik, 2001; Ashby and O’Brien, 2005; Poulin-Dubois et al., 2008). Nonetheless, we are predicting something different: that vocabulary development will be related to the *precision* of individual infants’ language-categorization link, not to infants’ categorization more generally. Our task was designed to assess this link specifically.

## Materials and Methods

### Participants

Twenty-four 12-month-old infants (*M* = 11.99 months; range = 11.57–12.50 months; 12 Female) were recruited from Evanston, IL, USA to participate. An additional 10 infants were run but excluded and replaced for fussiness (*N* = 5), technical error (*N* = 3), parental interference (*N* = 1), or because the caregiver reported a vocabulary size that was >2.5 *SD* from the overall mean at 12 months (*N* = 1). Another six infants were excluded in the analyses at 18 months because their caregivers failed to complete the second vocabulary assessment. All infants were English-acquiring monolinguals (at least 75% exposure to English). Northwestern University’s Internal Review Board approved the recruitment and experimental methods of this study (#STU00013062-MOD0004).

### Procedure

#### Phase 1 (12 Months)

After consenting to the study, caregivers completed the MacArthur-Bates Communicative Development Inventory (Words and Gestures short form; hereafter MCDI) and then accompanied their infants to a testing room for the categorization task. This task included two phases (see **Figure 1**). During the Familiarization phase, each infant saw eight line-drawn, colored images depicting distinct members of a single object category (either dinosaurs or fish). These were presented one at a time for 20 s each, each in conjunction with a distinctly different label produced by a female using infant-directed speech (e.g., *Look at the* /dov/*! Do you see the* /dov/*?*). These labels, designed to differ both in syllabic structure (either consonant–vowel–consonant or vowel–consonant–consonant) and in phonemes, were discriminable by 12-month-old infants^1^ (Werker and Curtin, 2005). Labeling occurred when the images first appeared and then again after 10 s. During the Test phase, a colorful spinning wheel appeared at the center of the screen to attract infants’ attention. Next, two new images appeared: a new member of the now-familiar category (e.g., another dinosaur) and a member of a novel category (e.g., a fish). These were presented side-by-side and in silence for 20 s.

#### Phase 2 (18 Months)

Six months later, when the infants were 18 months, parents were contacted via email and asked to complete the MCDI again. Parents were contacted up to three times.

## Results

We first classified each infant as having either a precise or broad link, then asked whether link specificity is related to vocabulary size at either 12 or 18 months.

### Identifying the Precision of Infants’ Links

For each infant, we calculated a novelty preference score (accumulated time looking toward the novel test object/accumulated time looking toward both the novel and familiar test objects) based on infants’ first 10 s of looking during the Test phase (as in Fulkerson and Waxman, 2007; Ferry et al., 2010). As predicted, 12-month-olds failed, as a group, to form object categories: their performance at test (*M* = 0.52, *SD* = 0.12) did not differ from chance, *t*(23) = 0.64, *p* = 0.53, an outcome that contrasts clearly to 12-month-olds’ performance with the very same set of objects when a single novel word is applied consistently to each (Fulkerson and Waxman, 2007). Infants’ performance in the Variable Word condition here replicated Waxman and Braun’s (2005) central finding that infants fail to form object categories when objects are presented in conjunction with different names.

**FIGURE 2:**
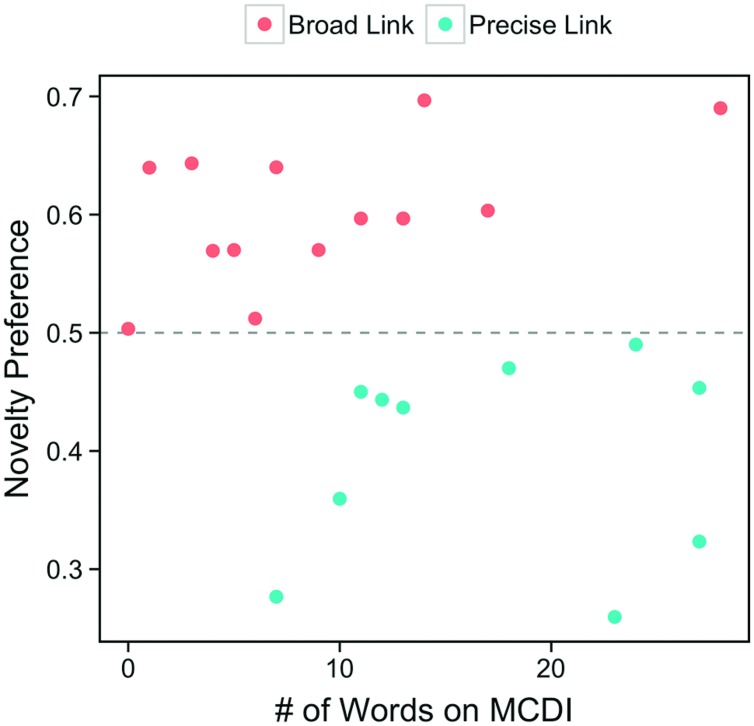
**Infants’ novelty preference scores and total receptive vocabulary counts on the MCDI at 12 months.** The color of each point indicates whether this infant was classified as having a “broad” link between language and categories (because they showed a novelty preference at test) or a “precise” link between language and categories (because they failed to show a novelty preference at test).

We then assigned infants to one of two groups, based on their performance at test (see **Figure 2**). Infants demonstrating a novelty preference were assigned to the *broad* link (*N* = 14) group. Infants demonstrating chance (0.5) or familiarity preferences were assigned to the *precise* link *(N* = 10) group^2^. Because these two groups were unbalanced and heterogeneous in variance, we performed Welch’s *t*-tests for all group comparisons.

### Is there a Relation between the Precision of Infants’ Link and Vocabulary Size (**Figure 3**)?

For each infant, we counted the total number of words in their vocabulary using the MCDI. We also split these totals into noun and non-noun subsets to assess whether any observed differences in total vocabulary were driven by their knowledge of nouns (which often label object categories in early vocabularies) or by their knowledge of all kinds of words (including verbs, animal sounds, greetings, and others on the MCDI). At 12 months, our analyses focused on their receptive vocabularies alone because infants at this age produce very few words. At 18 months, we analyzed both their receptive and productive vocabularies.

**FIGURE 3:**
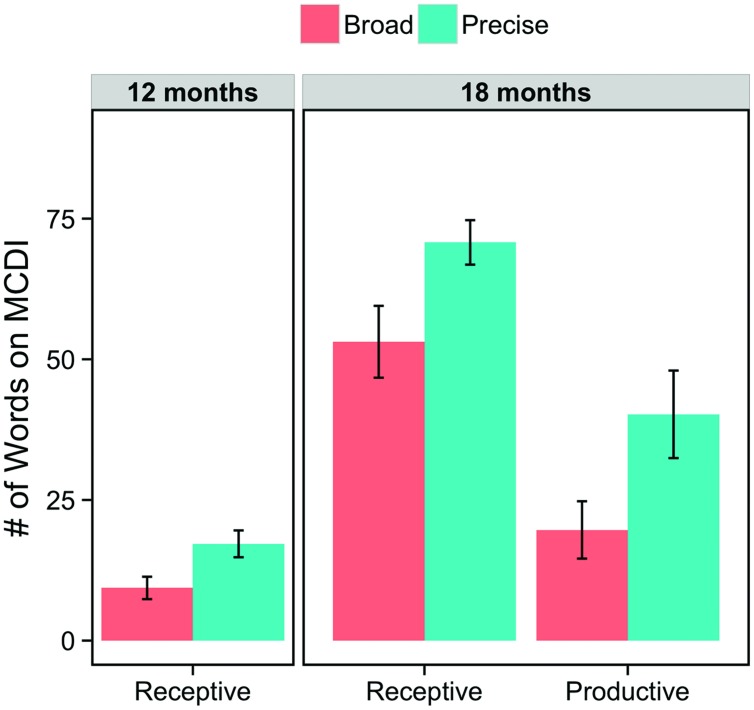
**Infants’ receptive and productive vocabularies at 12 and 18 months, grouped by the precision of their link.** Infants with a broad link between labels and categories had consistently smaller vocabularies than infants with a precise link.

#### Vocabulary Differences at 12 Months

An analysis of infants’ receptive MCDI scores at 12 months revealed that infants with precise links had higher receptive vocabularies (*M* = 17.20, *SD* = 7.54) than did infants with broad links (*M* = 9.36, *SD* = 7.44), *t*(19.38) = 2.53, *p* = 0.020. This difference held up if we considered only nouns (Precise: *M* = 10.70, *SD* = 5.48; Broad: *M* = 5.14, *SD* = 3.92), *t*(15.36) = 2.75, *p* = 0.015, but was only marginal when we considered only non-nouns (Precise: *M* = 6.50, *SD* = 2.55; Broad: *M* = 4.21, *SD* = 3.93), *t*(21.87) = 1.72, *p* = 0.099. These results reveal that, at 12 months, the precision of infants’ link between language and categories relates to vocabularies and, further, that this relation is positive: Those infants with a more precise link have larger vocabularies. Furthermore, this relation with vocabulary appears to be strongest when considering infants’ noun vocabularies alone.

#### Vocabulary Differences at 18 Months

The vocabulary differences between precise- and broad-link infants persisted from 12 to 18 months. Considering first their receptive vocabularies, once again, infants with a precise link between language and categories had significantly larger total vocabularies (*M* = 70.78, *SD* = 11.85) than those with a broad link (*M* = 53.11, *SD* = 19.16), *t*(13.34) = 2.35, *p* = 0.035. This difference also held when considering only nouns (Precise: *M* = 45.00, *SD* = 4.06; Broad: *M* = 33.89, *SD* = 10.42), *t*(10.38) = 2.98, *p* = 0.013, but was not reliable for non-nouns (Precise: *M* = 25.78, *SD* = 9.54; Broad: *M* = 19.22, *SD* = 9.60), *t*(15.99) = 1.45, *p* = 0.17.

Finally, considering their productive vocabularies, we see a similar pattern of results. Infants with a precise link had larger productive vocabularies (*M* = 40.22, *SD* = 23.34) than those with a broad link (*M* = 19.67, *SD* = 15.30), *t*(13.80) = 2.21, *p* = 0.045. In contrast to the previous comparisons, however, this difference was only marginal considering nouns alone (Precise: *M* = 24.56, *SD* = 14.20; Broad: *M* = 13.56, *SD* = 10.83), *t*(14.95) = 1.85, *p* = 0.085, yet was reliable considering non-nouns alone (Precise: *M* = 15.67, *SD* = 9.35; Broad: *M* = 6.11, *SD* = 5.41), *t*(12.83) = 2.65, *p* = 0.020.

### Ruling Out Alternative Interpretations and Potential Confounds

In a subsequent set of analyses, we sought to rule out alternative explanations for the relation between specificity of link and vocabulary size.

#### Age

One possibility is that infants who were classified as having a precise link were simply older than ‘broad’ infants and, therefore, had larger vocabularies. Indeed, although participants were all within 2 weeks of their first birthday, there was nonetheless a correlation between vocabulary size and age, *r*(22) = 0.43, *p* = 0.036. We therefore fit a series of linear models including Age and Link (Precise or Broad) as continuous and discrete variables, respectively, predicting infants’ total vocabulary at 12 and 18 months. These analyses indicated that Link independently predicted vocabulary sizes even when controlling for age: When predicting total vocabulary sizes at 12 months, we saw reliable effects of both Link [β = –7.78, *F*(1,21) = 7.98, *p* = 0.011] and Age [β = 10.74, *F*(1,21) = 6.39, *p* = 0.020], with this model accounting for 41% of the total variance in receptive vocabulary sizes, *p* = 0.0042. At 18 months, we once again found a reliable effect of Link [β = –16.27, *F*(1,15) = 4.71, *p* = 0.046], though in this case no reliable effect of Age [β = 10.07, *F*(1,21) = 0.58, *p* = 0.46], with the model accounting for 28% of variance in receptive vocabulary sizes, *p* = 0.081. The same pattern emerged in a model using production as the dependent variable.

#### Attention and Habituation during Familiarization

Another possibility is that infants’ vocabulary sizes related to their performance during the familiarization phase of the categorization task. For example, infants with larger vocabularies may have been less attentive and thus failed to form the category. Or perhaps they habituated more quickly in the task and were less attentive by the critical test phase. To test these possibilities, we looked for a correlation between vocabulary size and the proportion of time spent looking during familiarization (indexing total attention) as well as the difference in looking between the last four familiarization trials and the first four familiarization trials (indexing habituation). In both cases, these correlations were unreliable: Neither infants’ total attention [*r*(22) = –0.05, *p* = 0.81; Spearman’s ρ = –0.027, *p* = 0.90] nor their habituation [*r*(22) = –0.30, *p* = 0.16; ρ = –0.29, *p* = 0.17] during familiarization correlated with vocabulary size.

Therefore, the observed relation between vocabulary size and the specificity of the link to categorization at 12 and 18 months cannot be fully attributed to differences between infants’ age, attention, or habituation during the familiarization phase.

## Discussion

These results provide the first demonstration that the precision of an individual infant’s language-categorization link at 12 months is related to that infant’s vocabulary size. At 12 months, infants who had transitioned from a broad to the more precise language-category link – infants who did not form an object category when each member was introduced with a distinct noun – had larger vocabularies than did infants who still exhibited the broader language-category link. Remarkably, this relation remained stable through 18 months of age, as was evident in analyses of both vocabulary production and comprehension. Finally, although this relation was statistically stronger when considering nouns than non-nouns, we interpret this outcome with caution because it may simply reflect the relative scarcity of non-nouns in infants’ early vocabularies (Dale and Fenson, 1996).

Nevertheless, because the data we present here are correlational, the causal direction of the relation remains an open question. One possibility is that increases in vocabulary size leads to increases in the precision of the link between language and object categories (see Byers Heinlein and Werker, 2009 for a similar argument). A second possibility is that increases in the precision of the link catalyze future vocabulary growth. That is, infants who tune into object labels as specific guides to category have an advantage in learning new words, perhaps by better focusing their attention on discovering referent categories. Finally, a third possibility is that both the precision of infants’ link and their vocabulary growth relate to a third variable; candidate third variables include differences in infants’ (1) interest in speech (Vouloumanos and Werker, 2004, 2007; Shultz and Vouloumanos, 2010), (2) ability to segment and remember specific labels (Newman et al., 2006; Weill, 2011; Junge et al., 2012; Singh et al., 2012), (3) ability to discriminate words’ sounds (Tsao et al., 2004; Yeung et al., 2014), or (4) their amount of language input (Hart and Risley, 1995; Fernald and Marchman, 2011; Weisleder and Fernald, 2013).

In further research, it will also be important to examine links between language and other cognitive processes. In the first year, listening to language influences more than object categorization alone (Vouloumanos and Waxman, 2014); it also facilitates object individuation (Dewar and Xu, 2007, 2009), abstract rule learning (Marcus et al., 2012), and even basic associative learning (MacKenzie et al., 2011; Reeb-Sutherland et al., 2011). Moreover, these links reveal signatures of developmental tuning. For example, while 7-month-olds relate any kind of sounds to objects (Marcus et al., 2012), 12-month-olds are much more restrictive, limiting this role to labels (Woodward and Hoyne, 1999; Hollich et al., 2000; Fennell and Waxman, 2010; MacKenzie et al., 2011) and, even further, to labels that are phonotactically acceptable in their native language (MacKenzie et al., 2012; May and Werker, 2014). By examining advances in the precision with which infants link language to a range of cognitive capacities, we will gain important insights into these links’ roles in language development.

## Conflict of Interest Statement

The authors declare that the research was conducted in the absence of any commercial or financial relationships that could be construed as a potential conflict of interest.

## Footnotes

1 A preliminary analysis, using a standard acoustic discrimination paradigm with a different set of infants, revealed that 12-month-olds do discriminate among each of the stimuli presented here.

2 As a group, familiarity preferences are thought to index slow or partial learning while novelty preferences index more efficient or complete learning (Colombo and Bundy, 1983; Roder et al., 2000). We know from prior work with these exact stimuli at this age that infants who succeed in forming the category show a novelty preference (Fulkerson and Waxman, 2007). Therefore, we interpret any infant showing a familiarity preference as “failing” to categorize. Whether this is a complete failure (i.e., driven by random noise or idiosyncratic preference) or a partial failure (i.e., driven by partial learning of the category) is ambiguous.
